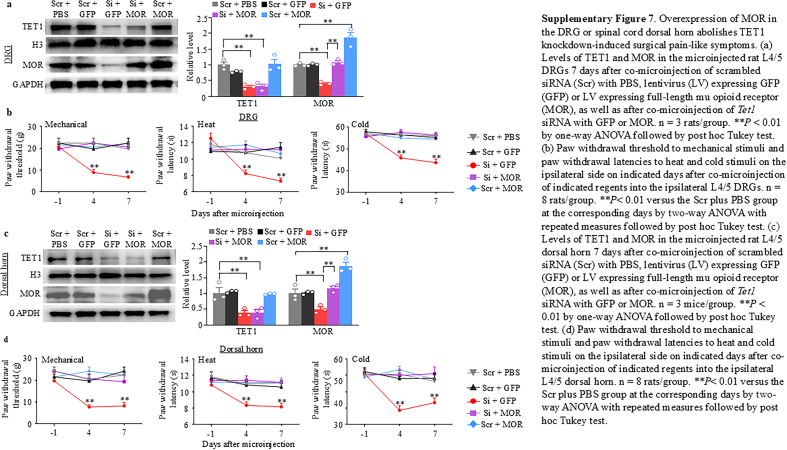# TET1 downregulation in the dorsal root ganglion and spinal cord is required for short-term sleep disturbance to delay surgical pain recovery

**DOI:** 10.1016/j.bja.2026.02.025

**Published:** 2026-05-05

**Authors:** Jing Cao, Po-Kai Wang, Bo Xu, Xiyao Gu, Zhiqiang Pan, Weihua Cai, Songxue Su, Zhixiao Li, Julia Delorenzo, Alex Bekker, Huijuan Hu, Yuan-Xiang Tao

**Affiliations:** 1 Department of Anesthesiology, New Jersey Medical School, Rutgers, The State University of New Jersey, Newark, NJ, USA; 2 Rutgers Graduate School of Biomedical Sciences, New Jersey Medical School, The State University of New Jersey, Newark, NJ, USA; 3 Department of Physiology, Pharmacology & Neuroscience, New Jersey Medical School, Rutgers, The State University of New Jersey, Newark, NJ, USA; 4 Department of Cell Biology & Molecular Medicine, New Jersey Medical School, Rutgers, The State University of New Jersey, Newark, NJ, USA

**Keywords:** TET1, mu opioid receptor, dorsal root ganglion, spinal cord dorsal horn, short-term sleep disturbance, postoperative pain

## Abstract

**Background:**

Sleep disturbance delays surgical pain recovery. This impact is associated with gene dysregulation in the dorsal root ganglion (DRG) and spinal dorsal horn (SDH). However, the mechanisms underlying this dysregulation remain unclear.

**Methods:**

Expression of ten–eleven translocation methylcytosine dioxygenase 1 (TET1) was examined in the DRG and SDH following plantar incision in rats subjected to short-term rapid eye movement sleep disturbance. A herpes simplex virus expressing *Tet1* mRNA (HSV-TET1) was microinjected into the ipsilateral L4 and L5 DRGs or SDH, and its effects on μ-opioid receptor (MOR) expression, TET1 binding to the *Oprm1* promoter, and promoter-associated 5-methylcytosine (5mC) and 5-hydroxymethylcytosine (5hmC) levels in microinjected regions were assessed. Pain behaviours were evaluated, and the effect of *Tet1* siRNA microinjection, with or without a lentivirus expressing *Oprm1* mRNA (LV-MOR), on MOR expression in microinjected DRGs and SDH and nociceptive threshold in naïve rats was assessed.

**Results:**

Short-term rapid eye movement sleep disturbance downregulated TET1 in the ipsilateral L4 and L5 DRGs and SDH and prolonged incisional pain. HSV-TET1 microinjection restored MOR expression, TET1 binding activity to the *Oprm1* promoter, and 5hmC levels at the promoter, while reducing 5mC accumulation, in microinjected L4 and L5 DRGs or SDH and prevented short-term rapid eye movement sleep disturbance-induced prolongation of incisional pain. Conversely, *Tet1* siRNA microinjection reduced MOR expression in microinjected L4 and L5 DRGs or SDH and induced nociceptive hypersensitivity, effects abolished by LV-MOR co-microinjection.

**Conclusions:**

TET1 downregulation is required for short-term sleep disturbance to delay surgical pain recovery, likely by reducing μ-opioid receptor expression in the dorsal root ganglion and spinal dorsal horn.

**Editor’s key points**

• Chronic postsurgical pain, affecting up to 10% of patients one year after surgery, remains a major clinical challenge.
• We show that short-term REM sleep deprivation (REMSD) after surgery prolongs pain by triggering neuronal hyperactivity in the dorsal root ganglion and spinal dorsal horn in a rat model of plantar incision.
• The mechanism involves specific downregulation of the epigenetic regulator TET1, which increases DNA methylation at the mu opioid receptor (MOR) promoter, thereby reducing MOR expression and impairing endogenous pain relief.
• Restoring TET1 levels in preclinical models reverses REMSD-induced pain prolongation, positioning TET1 as a promising, nonopioid therapeutic target for managing chronic postsurgical pain.

Chronic surgical pain, a pain syndrome after surgery, is a complex and debilitating health problem. Approximately half of surgical patients complain of surgical pain, of whom up to 10% report severe pain 1 yr after surgery.^1^^,^^2^ Chronic surgical pain significantly impacts the quality of life and results in the increase of pain-associated healthcare costs and a loss of productivity in surgical patients.^3^ Current medications, including opioids and non-opioid painkillers for this disorder’s treatment have limited effectiveness and cause unwanted side effects.^1^^,^^4^^,^^5^ The previous studies highlight associations between chronic surgical pain and several identified high-risk factors or predictors (e.g. sleep disturbances, anxiety, depression and preoperative pain) during perioperative periods.^6^, ^7^, ^8^, ^9^ Understanding how these factors impact the induction and maintenance of chronic surgical pain may provide insight into more efficient management of this disorder.

Sleep disturbance has been identified as one of the known risk factors impacting surgical pain. Chronic self-reported sleep disturbance before an operation significantly heightened surgical pain at rest in some surgical patients.^8^ The majority of surgical patients have short-term sleep disturbances throughout the pre and postoperative periods. For example, surgical patients who are hospitalised in the intensive care unit often complain of postoperative short-term sleep deprivation.^10^^,^^11^ Postoperative short-term sleep deprivation can result from a variety of factors ranging from surgical stress, a disruptive sleep environment (e.g. lighting, noise, and patient care activity), the medical illness itself, and disruptive medical treatments (e.g. medical ventilation, respiratory care, and drug therapies) and is positively correlated with surgical pain increase.^12^^,^^13^ Preclinical studies have also shown that pre and postoperative short-term rapid eye movement sleep disturbance (REMSD) exacerbated and prolonged postsurgical nociceptive hypersensitivity.^14^ These effects are likely attributed to the changes in gene expression in the dorsal root ganglion (DRG) and spinal cord dorsal horn (SDH).^15^^,^^16^ However, molecular mechanisms underlying these changes are still elusive.

It is well known that DNA methylation inhibits gene transcription. In mammalian cells, three types of functional DNA methyltransferases (DNMTs), DNMT1, DNMT3a and DNMT3b, have been identified. They transfer a methyl group to the 5^th^ position of a cytosine residue to catalyse cytosine to 5-methylcytosine (5mC), resulting in the repression of gene transcription.^17^ DNA methylation can also be removed by ten-eleven translocation methylcytosine dioxygenases (TETs), including TET1-3. The latter converts 5mC to 5-hydroxymethylcytosine (5hmC), leading to DNA demethylation and gene transcription.^18^^,^^19^ Therefore, gene transcriptional activity depends on the balance of the activity and expression between DNMTs and TETs.

In the present study, we showed that postoperative short-term REMSD delayed the recovery of surgical pain and specifically downregulated the expression of TET1, but not TET2, TET3, and DNMTs, in the ipsilateral L4 and L5 DRGs and SDH. This downregulation is responsible for the delay of surgical pain recovery by reducing the expression of the mu opioid receptor (MOR) in the DRG and SDH. TET1 is likely a potential target for the treatment of sleep disturbance-induced exacerbation and prolongation of surgical pain.

## Methods

### Animal preparations

Male Sprague-Dawley rats weighing 200–300 g were obtained from Charles River Laboratories (Wilmington, MA, USA). *Tet1*^f/f^ mice were provided generously by Anjana Rao from the La Jolla Institute for Allergy and Immunology (La Jolla, CA, USA). The rats and mice were housed in an animal facility with a standard 12-h light and dark cycle, with standard laboratory water and food pellets available *ad libitum*. Experiments were conducted with the approval of the Animal Care and Use Committee at Rutgers New Jersey Medical School and were consistent with the ethical guidelines of ARRIVE, the US National Institutes of Health and the International Association for the Study of Pain. All efforts were made to minimise animal suffering and to reduce the number of animals used. To minimise intra- and interindividual variability of behavioural outcome measures, animals were trained for 1–2 days before behavioural testing. The experimenters were blind to treatment conditions during behavioural testing.

### Incisional pain model

The incisional surgery was carried out with minor modifications as described.^14^^,^^20^ Rats were anaesthetised with 2 vol% isoflurane delivered through a nose cone. The plantar aspect of the left hind paw was prepared in a sterile manner with a 10% povidone-iodine solution. A 1-cm longitudinal incision was made with a number 11 blade through the skin and fascia of the plantar aspect of the foot, starting 0.5 cm from the proximal edge of the heel and extending toward the toes. The plantaris muscle was elevated and incised longitudinally. After haemostasis with gentle pressure, the skin was sutured with 4-0 nylon thread. The wound site was covered with a mixture of polymyxin B, neomycin, and bacitracin ointment. After surgery, the animals were allowed to recover in their cages. Typically, the wounds healed well within five to six days.

### Short-term REMSD procedure

The pedestal-over-water or flowerpot technique of short-term REMSD was conducted 2 h after incision, according to previous studies with minor modifications.^14^ Briefly, the rats were placed on a glass platform of 5.5 cm in diameter in the middle of a Plexiglas tank filled with water (25°C, 15 cm in depth) to 1 cm below the top surface of the platform. The occurrence of rapid eye movement sleep was disturbed by the muscular atonia accompanying the onset of rapid eye movement sleep, during which the body came into contact with the water, thus awakening the animal. These rats were maintained in the tank for 6 hr per day for 3 consecutive days during the daytime (11:00 to 17:00). The control rats were placed on a glass platform of 20 cm in diameter and 1 cm in height in the middle of the same tank without water.

### Dorsal root ganglion microinjection

DRG microinjection procedures were conducted following stereotaxically guided protocols established in our earlier publications.^21^, ^22^, ^23^, ^24^, ^25^ In brief, a midline incision was made in the lower lumbar back region, and the unilateral L4 and L5 DRGs were exposed. *Tet1* small interfering RNA (siRNA; 20 μM; Santa Cruz Biotechnology, Dallas, TX, USA; Supplementary Table 1), negative control siRNA (Supplementary Table 1; 20 μM; Santa Cruz Biotechnology), HSV or lentivirus (LV) was microinjected into the DRG (1 μl per DRG) with a glass micropipette (tip diameter 20–40 μm) connected to a Hamilton syringe. The pipette was removed 10 min after injection. The surgical field was irrigated with sterile saline, and the skin incision was closed with wound clips.

### Intraspinal cord microinjection

Spinal cord microinjection was carried out as described with minor modification.^26^^,^^27^ Briefly, under constant anaesthesia with isoflurane, unilateral laminectomy of thoracic vertebra 12 was conducted. After the spinal cord was exposed, the rat was placed in the stereotaxic frame, and the vertebral column was immobilised. The glass micropipette was positioned 200 mm lateral from the posterior median sulcus and 200 mm below the dorsal surface of the spinal cord at the level of the L4 and L5 spinal cord. The remaining procedure of microinjection and the dosage and volume of siRNAs, HSV or LV microinjected were similar to those in DRG microinjection described above. Two injection sites within a 1 mm interval along the posterior median sulcus were carried out. No impairment of motor function after intraspinal cord microinjection was observed.

### Behavioural tests

Mechanical, heat, cold and tail flick reflex tests, conditioned place preference (CPP) tests, and locomotor function tests were carried out as described.^21^, ^22^, ^23^, ^24^, ^25^ Mechanical, heat and cold tests were conducted at 1 h intervals.

Paw withdrawal thresholds in response to mechanical stimuli were measured with the up-down testing paradigm in rats and the frequency testing paradigm in mice. Briefly, rats or mice were placed in Plexiglas chambers on an elevated mesh screen. Von Frey filaments in log increments of force (rats: 0.407, 0.692, 1.202, 2.041, 3.63, 5.495, 8.511, 15.14 g; mice: 0.07 and 0.4 g) were applied to the plantar surface of the bilateral hind paws. For rats, the 2.041 g stimulus was applied first. If a positive response occurred, the next smaller von Frey hair was used; if a negative response was observed, the next larger von Frey hair was used. The test was terminated when (i) a negative response was obtained with the 15.14 g hair or (ii) three stimuli were applied after the first positive response. The paw withdrawal threshold was determined by converting the pattern of positive and negative responses to the von Frey filament stimulation to a 50% threshold value with a formula provided by Dixon.^28^ For mice, a rapid withdrawal of the paw was recorded as a positive response. Paw withdrawal frequency was calculated as the proportion of positive responses over 10 trials.

Paw withdrawal latencies to noxious heat were measured with a Model 336 Analgesic Meter (IITC Inc. Life Science Instruments, Woodland Hills, CA, USA). Rats or mice were placed in a Plexiglas chamber on a glass plate. Radiant heat through a hole from the lightbox was applied by aiming a beam of light through the glass plate to the middle of the plantar surface of each hind paw. When the animal lifted its foot, the light beam was turned off. The length of time between the start of the light beam and the foot lift was defined as the paw withdrawal latency. Each trial was repeated five times at 5 minute intervals for each side. A cut-off time of 20 s was used to avoid tissue damage to the hind paw.

Paw withdrawal latencies to noxious cold (0°C) were measured with a cold plate, which was set at 0°C. The duration between the placement of the hind paw and the animal lifting its hind paw, with or without paw licking and flinching, was defined as the paw withdrawal latency. Each trial was repeated three times at 10 min intervals for the paw on the ipsilateral side. A cut-off time (rats: 60 s; mice: 20 s) was used to avoid paw tissue damage.

A tail flick test was carried out to observe the effect of morphine analgesia. A tail-flick apparatus (Model 33B Tail Flick Analgesia Meter, IITC Life Science, Woodland Hills, CA, USA) with a radiant heat source connected to an automatic timer was used to assess the analgesic response. A cut-off time latency of 10 s was used to avoid tissue damage to the tail. Tail flick latencies were measured as the time required to induce a tail flick after applying radiant heat to the skin of the tail. The antinociceptive effects were expressed as the percentage of maximal possible analgesic effect (% MPAE): % MPAE = ([response latency-baseline latency] [cut-off latency-baseline latency]^-1^)×100%.

For the CPP test, an apparatus with two Plexiglas chambers (same size, different visual and textured cues) connected by a 4 cm×6 cm removable door (MED Associates Inc., St Albans, VT, USA) was used. Time spent in each chamber was monitored by photobeam detectors and automatically recorded in MED-PC IV CPP software. The animals were first preconditioned for 30 minute with full access to both chambers to habituate them to the environment. At the end of the preconditioning phase, the basal duration spent in each chamber was recorded within 15 min. The animals that spent >720 or <180 s in either chamber were excluded from further testing. The conditioning protocol was then performed for the following 3 days with the internal door closed. The animals were first injected intrathecally with saline (5 μl) specifically paired with one conditioning chamber in the morning for 30 min. About 6 h later, the animals were then injected intrathecally with lidocaine (0.4%, 5 μl) and placed in another chamber for 30 min. The order of the injections of saline and lidocaine was switched every day. On the test day, at least 20 hr after conditioning, the animals were placed in one chamber with free access to both chambers. The time spent in each chamber was recorded for 15 min. Difference scores for chamber preference were calculated by subtracting preconditioning preference time from test time spent in the lidocaine-paired chamber.

Locomotor function, including placing, grasping, and righting reflexes, was examined after the above-described behavioural tests. (1) Placing reflex: the hind limbs were placed slightly lower than the forelimbs, and the dorsal surfaces of the hind paws were brought into contact with the edge of a table. Whether the hind paws were placed on the table surface reflexively was recorded. (2) Grasping reflex: after the animal was placed on a wire grid, whether the hind paws grasped the wire on contact was recorded. (3) Righting reflex: when the animal was placed on its back on a flat surface, whether it immediately assumed the normal upright position was recorded. Each trial was repeated 5 times at 5 min intervals, and the scores for each reflex were recorded based on counts of each normal reflex.

### Plasmid constructs and virus production

Two segments of rat *Tet1* coding sequences were amplified separately from total RNA of rat DRG using the SuperScript III One-Step RT-PCR System with the Platinum *Taq* High Fidelity Kit (Invitrogen) and the primers (Supplementary Table 1). After nested PCR of each product using Platinum *Pfx* DNA Polymerase (Invitrogen, location?) and the primers (Supplementary Table 1), respectively, the PCR products were inserted into the pENTR-D-TOPO vector (Invitrogen) and validated by sequencing. Two plasmids harbouring each end of the coding sequences of rat *Tet1* were combined into one plasmid using *Kpn*I and *Asc*I restriction sites to get the full-length *Tet1* plasmid. The resulting vector was submitted to the Viral Gene Transfer Core at MIT for the LR recombination reaction and HSV p1005 package. The HSV-GFP used as a control was provided by Eric J. Nestler (Icahn School of Medicine at Mount Sinai, NY, USA). LV expressing full-length *Oprm1* mRNA (LV-MOR) or *Gfp* mRNA (LV-GFP) was purchased from Hunan Fenghui Biotechnology Co., Ltd. (city, China).

### Neuronal culture, transfection, and transduction

Primary DRG neuronal cultures and transfection were carried out as described.^21^, ^22^, ^23^, ^24^, ^25^ Briefly, four-week-old rats were killed with isoflurane, and all DRGs were collected in cold Neurobasal Medium (Gibco-ThermoFisher Scientific, location?) containing 10% fetal bovine serum (JR Scientific, Woodland, CA, USA), penicillin 100 units ml^−1^, and streptomycin 100 μg/ml (Quality Biological, Gaithersburg, MD, USA) and then treated with enzyme solution containing dispase 5 mg ml^−1^ and collagenase type I 1 mg ml^−1^ in Hanks’ balanced salt solution (HBSS) without Ca^2+^ and Mg^2+^ (Gibco-ThermoFisher Scientific). Dissociated cells were resuspended in mixed Neurobasal Medium and plated in a six-well plate pre-coated with 50 μg ml^−1^ poly-d-lysine (Sigma, St Louis, MO, USA). The cells were incubated at 95% O_2_, 5% CO_2_ and 37°C. One day later, siRNA (100 nM; Santa Cruz Biotechnology) or 2-10 μl of virus (titre ≥5×10^12^ tu μl^−1^) was added to each 2 ml well. The neurones were collected 3 days later.

### Reverse transcription (RT)-PCR assay

Quantitative real-time RT-PCR assay was conducted as described previously.^21^, ^22^, ^23^, ^24^, ^25^ Briefly, extracted RNA was treated with DNase I (New England Biolabs, Ipswich, MA, USA) and reverse-transcribed using the ThermoScript reverse transcriptase (Invitrogen-ThermoFisher Scientific, location?) and random hexamers. The template (1 μl) was amplified by real-time PCR using the primers listed in Supplementary Table 1 (Integrated DNA Technologies, location?). *Gapdh* mRNA was used as an internal control for normalisation. Each sample was run in triplicate in a 20 μL reaction with 250 nM forward and reverse primers, 10 μl of SsoAdvanced Universal SYBR Green Supermix (Bio-Rad Laboratories, Hercules, CA, USA) and cDNA 20 ng. Reactions were performed in a BIO-RAD CFX96 real-time PCR system. Ratios of RNA levels in other treated groups to RNA level in the control group were calculated using the ΔCt method (2^-DDCt^). All data were normalised to *Gapdh* mRNA.

For single-cell real-time RT-PCR, freshly dissociated rat DRG neurones or SDH neurones were first prepared as described.^27^ Briefly, 4 h after plating, a single living neurone was collected under an inverted microscope fitted with a micromanipulator and microinjector and placed in a PCR tube with 5–10 μl of cell lysis buffer (Signosis, Sunnyvale, CA, USA). After centrifugation, the supernatants were collected. The remaining real-time RT-PCR procedure was carried out according to the manufacturer’s instructions with the single-cell real-time RT-PCR assay kit (Signosis). All primers used are listed in Supplemental Table 1. After amplification, PCR products were separated on a 2.0% agarose gel containing 0.025% ethidium bromide; bands were visualised using ChemiDoc™ XRS + Imaging Systems (Bio-Rad Laboratories, Location?).

### Chromatin immunoprecipitation (ChIP) assay

The TET1 ChIP assay was conducted using the EZ ChIP Kit (Upstate-EMD Millipore, Darmstadt, Germany) as described^21^, ^22^, ^23^, ^24^, ^25^ with minor modifications. Briefly, the homogenates from the L4 and L5 DRGs were crosslinked with 1% formaldehyde for 10 minute at room temperature. The reaction was terminated by the addition of 0.25 M glycine. After centrifugation, the collected pellet was lysed by sodium dodecyl sulfate lysis buffer with protease inhibitor cocktail and sonicated until the DNA was broken into fragments with a mean length of 200 to 1,000 bp. After the samples were pre-cleaned with protein G agarose, they were respectively subjected to immunoprecipitation overnight with 4 μg of rabbit TET1 antibody (Abcam, location?) or 4 μg of normal rabbit serum overnight at 4°C. The input (10%–20% of the sample for immunoprecipitation) was used as a positive control. The DNA fragments were purified and identified using PCR real-time PCR with the primers listed in Supplementary Table 1.

For the 5hmC and 5mC ChIP assay, genomic DNA was extracted from the ipsilateral L4 and L5 DRG or ipsilateral L4 and L5 SDH using the DNeasy Blood & Tissue Kit (Qiagen, location?) according to the manufacturer’s instructions and then sonicated to 100–500 bp fragments. Immunoprecipitation for 5hmC was performed using the Thermo Scientific EpiJET 5-hmC Enrichment Kit (Thermo Scientific). Briefly, 5hmC in 500 ng fragmented genomic DNA was modified by the linker through modification enzyme. After being purified, the 40 μl DNA sample with 5hmC-linker was chemically modified by biotin moiety using biotin 50 μl reagent at 50°C for 5 min and then mixed with 20 μl streptavidin-coated magnetic beads and 40 μl streptavidin binding buffer. Finally, DNA with 5hmC was separated from the beads after being washed and incubated at 70°C for 5 min. Immunoprecipitation for 5mC was carried out using the Methylated-DNA IP Kit (Zymo Research). Briefly, 160 ng fragmented DNA in 50 μl DNA denaturing buffer was denatured at 98°C for 5 min. The 250 μl MIP buffer containing 15 μl of ZymoMag Protein A and 1.6 μl mouse anti-5-methylcytosine was added at 37°C for 1 h on a rotator. After the beads were suspended with 500 μl MIP buffer, DNA was precipitated and resuspended in 200 μl of water for real-time PCR analysis as described above. The no-enzyme group in the reaction was used as a negative control. Equal genomic DNA was used to be input control. Seven (7) sets of primers in the *Opmr1* promoter (Supplementary Table 1) were used to amplify different regions of the 5hmC- or 5mC-immunoprecipitated *Opmr1* promoter, respectively. The quantitative analysis for 5hmC or 5mC was performed as described.^29^

### Single- or double-labelled immunohistochemistry

Rats were anaesthetised with isoflurane and perfused with 4% paraformaldehyde in 0.1 M phosphate-buffered saline (PBS, pH 7.4). L4 and L5 DRGs and the spinal cord were removed, post-fixed, and dehydrated before cryosectioning at 20 μm. Approximately four sets of sections (10–12 sections DRG^−1^ rat^−1^ and 14–16 sections spinal cord^−1^ rat^−1^) were collected from each DRG or spinal cord by grouping every fourth serial section. Each set of sections was used for the following single- or double-labelled immunofluorescent staining. Briefly, after the sections were blocked for 1 h at room temperature in 0.01 M PBS containing 10% goat serum and 0.3% Triton X-100, they were incubated with the following primary antibodies or the reagents over one or two nights at 4°C. The antibodies and reagents include rabbit anti-TET1 (1:800, Abcam), mouse anti-TET1 (1:800, Abcam), mouse anti-NF200 (1:500, Sigma, St Louis, MO), biotinylated IB4 (1:100, Sigma), mouse anti-CGRP (1:50, Abcam), mouse anti-NeuN (1:50, GeneTex, Irvine, CA), guinea pig anti-MOR (1:1,000, EMD Millipore), mouse anti-GS (1:100, Abcam), mouse anti-GFAP (1:500, CST), or mouse anti-OX42 (1:200, EMD Millipore). The sections were then incubated with either goat anti-rabbit antibody conjugated to Cy3 (1:200, Jackson ImmunoResearch, West Grove, PA, USA), goat anti-mouse antibody conjugated to Cy2 (1:200, Jackson ImmunoResearch), goat anti-guinea pig antibody conjugated to Cy3 (1:200, Jackson ImmunoResearch), or avidin labelled with FITC (1:200, Sigma) for 2 hr at room temperature. Control experiments included substitution of the normal mouse or rabbit serum for the primary antiserum and omission of the primary antiserum. All immunofluorescence-labelled images were examined using a Leica DMI4000 fluorescence microscope and captured with a DFC365FX camera (Leica, city?, Germany). The numbers of positively and negatively labelled neurones per section were manually counted in the DRG, and the percentage of positively labelled neurones per section was calculated by dividing the number of positively labelled neurones by the total number of labelled and unlabelled neurones. Immunofluorescent staining density in laminae I–II and laminae III–VI of SDH was measured using NIH Image J software.

### Western immunoblotting assay

Two unilateral rat DRGs were pooled to achieve sufficient proteins. The tissues were homogenised, and the cultured cells were ultrasonicated in chilled lysis buffer (10 mM Tris, 1 mM phenylmethylsulphonyl fluoride, 5 mM MgCl2, 5 mM EGTA, 1 mM EDTA, 1 mM DTT, 40 μM leupeptin, 250 mM sucrose). After centrifugation at 4°C for 15 min at 1000 g, the supernatants were collected for cytosolic proteins and the pellets for nuclear proteins. The contents of the proteins in the samples were measured using the Bio-Rad protein assay (Bio-Rad), and then the samples were heated at 99°C for 5 min and loaded onto a 4%–15% stacking plus 7.5% separating SDS-polyacrylamide gel (Bio-Rad). The proteins were then electrophoretically transferred onto a polyvinylidene difluoride membrane (Bio-Rad). After the membranes were blocked with 3% non-fat milk in Tris-buffered saline containing 0.1% Tween-20 for 1 h, the following primary antibodies were used: rabbit anti-TET1 (1:1000, Abcam), rabbit anti-MOR (1:500, Neuromics, Minneapolis, MN), rabbit anti-GAPDH (1:1000, Santa Cruz), rabbit anti-H3 (1:1000, Santa Cruz), mouse anti-GFAP (1:1000, Abcam), rabbit anti-p-ERK1 and 2 (1:1000, CST), and rabbit anti-ERK1 and 2 (1:1000, CST). The proteins were detected by horseradish peroxidase–conjugated anti-mouse or anti-rabbit secondary antibody (1:3000, Jackson ImmunoResearch) and visualised by western peroxide reagent and luminol-enhancer reagent (Clarity Western ECL Substrate, Bio-Rad) and exposure by ChemiDoc XRS and System with Image Lab software (Bio-Rad). The density of blots was quantified with densitometry using Image Lab software (Bio-Rad). The relative density value from each treatment group was determined by dividing the optical density value by the value of the corresponding control group after each group was normalised to its corresponding GAPDH (for cytosol protein) or H3 (for nucleus protein) density.

### Electrophysiological recording

The acute disassociated DRG neurones from adult rats were cultured as described above. The action potential (AP) was recorded with the use of the whole-cell current clamp. The coverslips were placed in the chamber and perfused with the extracellular solution consisting of (in mM) NaCl 140, KCl 4, CaCl_2_ 2, MgCl_2_ 2, HEPES 10 and glucose 5, with pH adjusted to 7.38 by NaOH. The intracellular pipette solution contained (in mM) KCl 135, Mg-ATP 3, Na_2_ATP 0.5, CaCl_2_ 1.1, EGTA 2 and glucose 5; pH was adjusted to 7.38 with potassium hydroxide, and osmolality was adjusted to 300 mOsm with sucrose. The resting membrane potential was taken 3 min after a stable recording was first obtained. To evoke AP, we delivered depolarising currents from 100 to 1400 pA (100 pA increment, 200 ms duration). The AP threshold was defined as the first point on the rapidly rising phase of the spike at which the change in voltage exceeded 50 mV ms^−1^. The membrane potential was held at the existing resting membrane potential during the current injection. The AP amplitude was measured between the peak and the baseline, and the AP overshoot was measured between the AP peak and 0 mV. To measure the membrane input resistance, each cell was delivered with a series of hyperpolarising currents, 200 ms duration delivered in steps of 100 pA from 200 pA to 2000 pA and obtained the membrane input resistance from the slope of a steady-state I–V plot. The afterhyperpolarisation amplitude was measured between the maximum hyperpolarisation and the final plateau voltage. The data were stored on a computer by a DigiData 1500 interface and were analysed by the pCLAMP 10.4 software package (Molecular Devices, location?). All experiments were performed at room temperature.

### Statistical analysis

For *in vitro* experiments, the cells were evenly suspended and then randomly distributed in each well tested. For *in vivo* experiments, the animals were randomly assigned to various treatment groups. All the results are given as mean (SEM). Data were analysed with a two-tailed, paired Student *t*-test and a one-way or two-way anova. When anova showed a significant difference, pairwise comparisons between means were tested by the *post hoc* Tukey method (SigmaPlot 12.5, San Jose, CA, USA). Significance was set at *P*<0.05.

## Results

### Short-term sleep deprivation produces cellular hyperactivity in the DRG and dorsal horn and exacerbates and prolongs surgical pain

**Fig 1:**
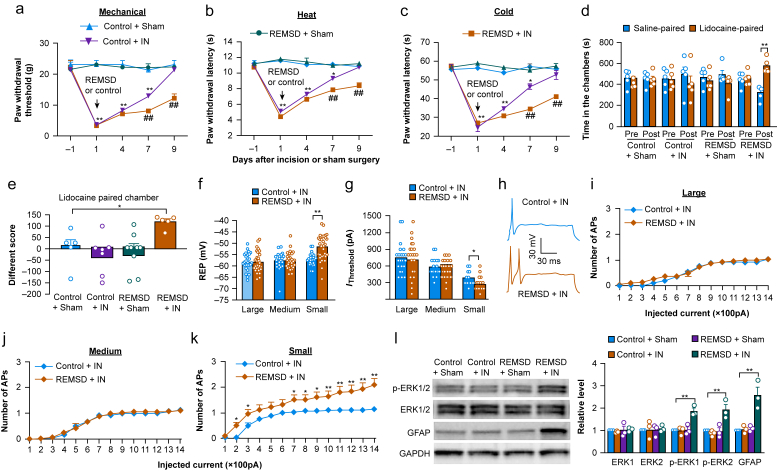
Short-term sleep deprivation produces cellular hyperactivity in the DRG and dorsal horn and exacerbates and prolongs surgical pain in rats. (a–c) Paw withdrawal threshold to mechanical stimulation (a) and paw withdrawal latency to heat (b) and cold (c) stimuli on the ipsilateral side on indicated days post-incision (IN) or sham surgery in rats with REMSD or control post-treatment. *n*=8 rats group^−1^. *^∗^P*<0.05, *^∗∗^P*<0.01 *vs* the control sham rats at the corresponding time point. *^##^P*<0.01 *vs* the control incisional rats at the corresponding time point. A three-way anova with repeated measures followed by a *post hoc* Tukey test. (d, e) Spontaneous ongoing pain as assessed by the CPP paradigm on day 9 post-incision (IN) or sham surgery in rats with REMSD or control post-treatment. *n*=8 rats group^−1^. *^∗^P*<0.05, *^∗∗^P*<0.01 by two- (e) or a three (d)-way anova with repeated measures followed by post hoc Tukey test. (f, g) Rest membrane potentials (f) and rheobases (g) in large, medium and small neurones from the ipsilateral L4 and L5 DRGs on day 9 postincision in rats with control or REMSD post-treatment. *n*=32 large, 30 medium and 31 small DRG neurones in REMSD plus incision (IN) group (8 rats). *n*=25 large, 24 medium and 26 small DRG neurones in the control plus incision group (7 rats). *^∗^P*<0.05, *^∗∗^P*<0.01 by 2-tailed unpaired Student *t* test. (h) Representative traces of evoked action potentials in small DRG neurones on day 9 postincision in rats with control or REMSD post-treatment. (i-k) Number of evoked action potentials after application of different currents as indicated on day 9 postincision in rats with control or REMSD post-treatment. Number of recorded neurones and rats used were the same as in f and g. *^∗^P*<0.05, *^∗∗^P*<0.01 *vs* the control plus incision group at the corresponding stimulation intensity by two-way anova with repeated measures followed by post hoc Tukey test. (l) Levels of p-ERK1 and 2, ERK1 and 2 and GFAP in the ipsilateral L4 and L5 SDH on day 9 post-incision (IN) or sham surgery in rats with control or REMSD post-treatment. *n*=3 rats group^−1^. *^∗∗^P*<0.01 by two-way anova followed by *post hoc* Tukey test.

Consistent with previous studies,^14^^,^^20^ incision-induced mechanical allodynia and heat and cold hyperalgesia reached a peak on day 1, lasted for 4 to 7 days and completely disappeared on day 9 after incision on the ipsilateral side of control rats (Fig. 1a–c). The rats exposed to short-term sleep deprivation caused by REMSD for 6 hr daily for 3 consecutive days after incision displayed exacerbated and prolonged mechanical allodynia and heat and cold hyperalgesia on the ipsilateral side on days 7 and 9 post-incision (Fig. 1a–c). In particular, significant nociceptive hypersensitivity was still observed on day 9 post-incision in these rats (Fig. 1a–c). Moreover, the REMSD-treated incisional rats exhibited the evoked stimulation-independent spontaneous pain, as shown by the significant preference for the lidocaine-paired chamber on day 9 post-incision (Fig. 1d and e). The control incisional rats, control sham rats and REMSD-treated sham rats revealed no marked preference for either chamber (Fig. 1d and e).

The REMSD-induced exacerbation and prolongation of incisional pain aforementioned indicate cellular hyperactivation in the DRG and SDH on day 9 post-incision. To this end, we carried out whole-cell current-clamp recording in acutely dissociated neurones from the ipsilateral L4 and L5 DRGs of control rats or REMSD-treated rats on day 9 post-incision and recorded DRG neuronal hyperexcitability. Compared with control incisional rats, small, but not medium and large, DRG neurones from the REMSD-treated incisional rats showed significant depolarisation by 5.7 mV in resting membrane potential (Fig. 1f) and reduction by 21% in the rheobase (Fig. 1g). Moreover, the number of action potentials evoked by injected currents was markedly increased in small, but not medium and large, DRG neurones from the REMSD-treated rats compared with those in the control rats on day 9 post-incision (Fig. 1h–k). Neither membrane input resistance nor remaining AP parameters (including amplitude, overshoot, threshold, and afterhyperpolarisation) of DRG neurones differed between these two groups (Supplementary Table 2). In addition, REMSD-treated incisional rats exhibited cellular hyperactivity in the dorsal horn as evidenced by the fact that the levels of p-ERK1 and 2 (a marker for neuronal hyperactivity) and GFAP (a marker for astrocyte), but not total ERK1 and 2, were dramatically increased in the ipsilateral L4 and L5 dorsal horn from the REMSD-treated rats 9 days post-incision (Fig. 1l). As predicted, the control incisional rats, control sham rats or REMSD-treated sham rats displayed basal levels of these markers in the dorsal horn on day 9 post-incision or sham surgery (Fig. 1l). Taken together, these findings suggest that short-term sleep deprivation leads to cellular hyperactivity in the DRG and dorsal horn, resulting in exacerbation and prolongation of postsurgical pain.

### Short-term sleep deprivation downregulates TET1 in the DRG and dorsal horn neurones after surgery

**Fig 2:**
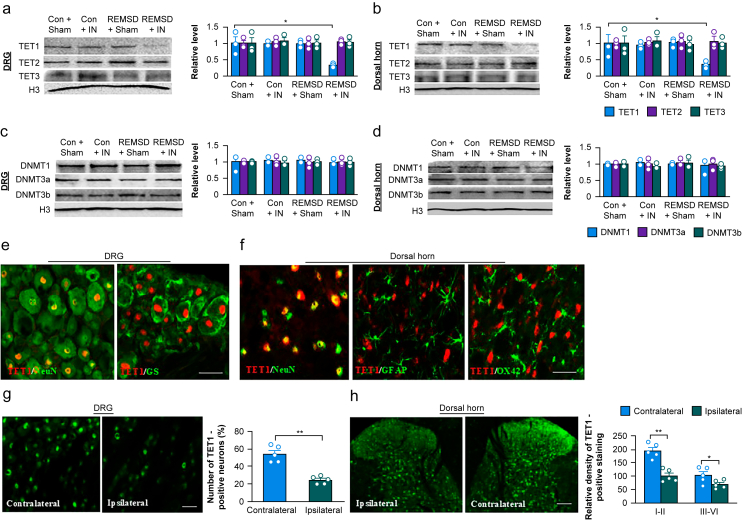
Short-term sleep deprivation downregulates TET1 in the DRG and dorsal horn neurones of rats after surgery. (a, b) Levels of TET1, TET2 and TET3 in the ipsilateral L4 and L5 DRGs (a) and dorsal horn (b) on day 9 postincision (IN) or sham surgery in rats with control (Con) or REMSD post-treatment. *n*=3 repeats (6 rats for DRG and 3 rats for dorsal horn) group^−1^. *^∗^P*<0.05 by two-way anova followed by a *post hoc* Tukey test. (c, d) Levels of DNMT1, DNMT3a and DNMT3b in the ipsilateral L4 and L5 DRGs (c) and dorsal horn (d) on day 9 post-incision (IN) or sham surgery in rats with control or REMSD post-treatment. *n*=3 repeats (6 rats for DRG and 3 rats for dorsal horn) group ^−1^. Two-way anova followed by *post hoc* Tukey test. (e) TET1(red) co-localised with NeuN (green), but not with glutamine synthetase (GS; green) in the ipsilateral L5 DRG from naïve rats. Approximately 53% of DRG neurones were labelled by TET1. *n*=3 rats. Scale bar: 50 μm. (f) TET1(red) co-expressed with NeuN (green), but not with GFAP (green) and OX42 (green) in the ipsilateral L5 dorsal horn from naïve rats. *n*=3 rats. Scale bar: 50 μm. (g) Number of TET1-positive neurones in the ipsilateral and contralateral L5 DRG on day 9 postincision in rats with REMSD post-treatment. *n*=5 repeats (rats). *^∗∗^P*<0.01 by 2-tailed unpaired Student *t* test. Scale bar: 50 μm. (h) Relative density of TET1-positive staining in the ipsilateral and contralateral L5 dorsal horn (laminae I–II and laminae III–VI) on day 9 postincision in rats with REMSD post-treatment. *n*=5 repeats (rats). *^∗^P*<0.05, *^∗∗^P*<0.01 by two-tailed unpaired Student’s *t* test. Scale bar: 100 μm.

We next investigated how short-term REMSD plus incision delayed surgical pain recovery. Our previous studies suggest that the changes of gene expression in the DRG and dorsal horn from the REMSD-treated incisional rats contribute to short-term REMSD-induced exacerbation and prolongation of surgical pain.^14^^,^^20^ Epigenetic DNA methylation controlled by DNMTs (including DNMT1, 3a and 3b) and TETs (including TET1-3) represses gene expression.^30^ We found that TET1, but not TET2, TET3, DNMT1, DNMT3a and DNMT3b, was downregulated in the ipsilateral L4 and L5 DRGs and dorsal horn from the REMSD-treated rats on day 9 post-incision (Fig. 2a–d). The level of TET1 in the ipsilateral L4 and L5 DRGs and dorsal horn decreased by 65% and 67%, respectively, in the REMSD-treated incisional group compared with the corresponding control sham rats 9 days after surgery (Fig. 2a and b). REMSD-treated sham rats and control incisional rats exhibited no change in basal levels of TET1–3, DNMT1, DNMT3a and DNMT3b in the ipsilateral L4 and L5 DRGs and dorsal horn (Fig. 2a–d). As expected, the basal level of TET1 in the contralateral L4 and L5 DRGs and dorsal horn was not altered among all treated groups (Supplementary Fig. 1).

Furthermore, TET1 co-expressed with NeuN (a specific marker of neurone), but not GS (a specific marker of satellite cell), in the L4 and L5 DRGs (Fig. 2e). In the L4 and L5 dorsal horn, TET1 co-localised with NeuN but not GFAP (a specific marker of astrocytes) and OX42 (a specific marker of microglia) (Fig. 2f). These findings show neurone-specific expression of TET1 in the DRG and dorsal horn. In the L4 and L5 DRGs, approximately 53% of neurones were TET1-positive. Size distribution analysis showed that about 50% of them were small (<600 μm^2^ in sectional area), 35% medium (600–1200 μm^2^ in sectional area) and 15% large (>1200 μm^2^ in sectional area) (Supplementary Fig. 2a). Consistently, approximately 29% of TET1-labelled neurones were positive for CGRP, 47% for IB4 and 28% for NF200 (Supplementary Fig. 2b). More importantly, the number of TET1-labelled neurones in the L4 and L5 DRGs and dorsal horn on the ipsilateral side from the REMSD-treated group on day 9 post-incision were significantly reduced compared with the corresponding contralateral side (Fig. 2g and h). Collectively, these data suggest that TET1 downregulation in the DRG and dorsal horn is negatively correlated with short-term REMSD-induced exacerbation and prolongation of surgical pain and likely is involved in the short-term sleep deprivation-induced delay of postsurgical pain recovery.

### Rescuing TET1 downregulation in the DRG or dorsal horn attenuates the short-term sleep deprivation-induced delay of surgical pain recovery

**Fig 3:**
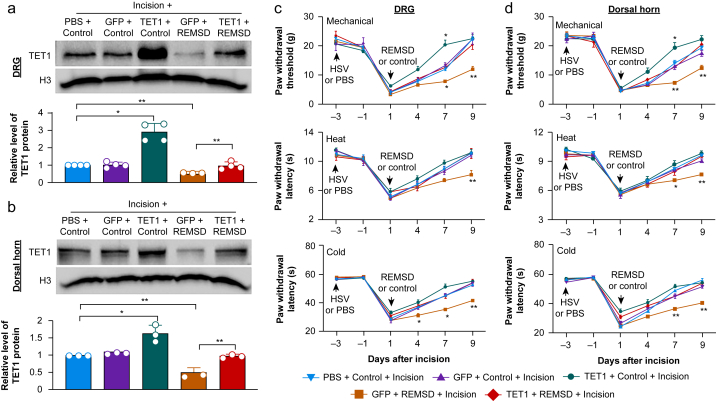
Rescuing TET1 downregulation in the dorsal root ganglion or dorsal horn attenuates the short-term sleep deprivation-induced delay of surgical pain recovery. (a, b) Level of TET1 in the ipsilateral L4 and L5 DRGs (a) and dorsal horn (b) on day 9 postincision in the control- or REMSD-treated rats with microinjection of PBS, HSV-GFP (GFP) or HSV-TET1 (TET1) into these two regions, respectively, 3 days before incision. n=3–4 repeats (rats) group^−1^. *^∗^P*<0.05, *^∗∗^P*<0.01 by one-way anova followed by *post hoc* Tukey test. (c, d) Paw withdrawal threshold to mechanical stimuli and paw withdrawal latencies to heat and cold stimuli on the ipsilateral side on indicated days postincision in the control- or REMSD-treated rats with microinjection of PBS, HSV-GFP (GFP) or HSV-TET1 (TET1) into the ipsilateral L4 and L5 DRGs (c) and dorsal horn (d), respectively, 3 days before incision. *n*=8 rats group^−1^. *^∗^P*<0.05, *^∗∗^P*<0.01 *vs* the PBS plus incision control rats at the corresponding time point by two-way anova with repeated measures followed by *post hoc* Tukey test.

To examine the role of TET1 downregulation in the DRG and dorsal horn in the short-term sleep deprivation-induced delay of surgical pain, we overexpressed TET1 in these two regions through microinjection of HSV expressing full-length *Tet1* mRNA (HSV-TET1). HSV expressing the enhanced green fluorescent protein (HSV-GFP) and PBS were used as the controls. Pre-microinjection of HSV-TET1, but not HSV-GFP and PBS, into the ipsilateral L4 and L5 DRGs or dorsal horn from the REMSD-treated group completely reversed a reduction of TET1 in the corresponding region on day 9 postincision (Fig. 3a and b). Unlike the HSV-GFP-microinjected incisional rats that underwent REMSD, the HSV-TET1-microinjected rats with REMSD treatment did not display exacerbated and prolonged mechanical allodynia and heat and cold hyperalgesia on day 7 or day 9 post-incision on the ipsilateral side (Fig. 3c and d). In addition, they did not show significant increases in p-ERK1 and 2 and GFAP levels in the ipsilateral L4 and L5 dorsal horn on day 9 postincision (Supplementary Fig. 3). In the HSV-TET1-microinjected control rats that received an incision, basal levels of TET1 in the ipsilateral L4 and L5 DRGs and dorsal horn on day 9 post-surgery were increased by 2.9 fold and 1.6 fold, respectively, compared with the corresponding PBS-microinjected control rats that underwent an incision (Fig. 3a and b). These TET1-increased rats displayed a significant elevation in paw withdrawal threshold to mechanical stimulation on day 7 after surgery (Fig. 3c and d). As expected, HSV-GFP microinjection in control rats that underwent an incision did not affect the basal level of TET1 in the ipsilateral L4 and L5 DRGs and dorsal horn and the development and recovery of incision-induced nociceptive hypersensitivity (Fig. 3c and d). All treated rats showed basal paw withdrawal responses to mechanical and heat stimuli on the contralateral side (Supplementary Fig. 4) and normal locomotor activities (Supplementary Table 3).

### Knocking down TET1 in the DRG or dorsal horn produces surgical pain-like symptoms

**Fig 4:**
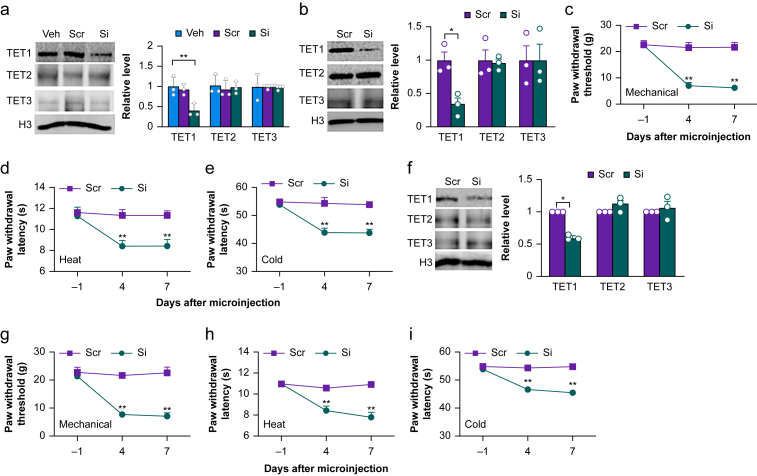
Knock-down of TET1 in the dorsal root ganglion or dorsal horn produces surgical pain-like symptoms. (a) Levels of TET1, TET2 and TET3 in the cultured DRG neurones with the treatments of vehicle (Veh), control scrambled siRNA (Scr) or *Tet1* siRNA (Si). *n*=3 biological repeats group^−1^. *^∗∗^P*<0.01 by one-way anova followed by *post hoc* Tukey test. (b) Levels of TET1, TET2 and TET3 in the microinjected rat L4 and L5 DRGs 7 days after microinjection of control scrambled siRNA (Scr) or *Tet1* siRNA. *n*=3 rats group^−1^. *^∗^P*<0.05 by two-tailed unpaired Student *t* test. (c–e) Paw withdrawal threshold to mechanical stimuli (c) and paw withdrawal latencies to heat (d) and cold (e) stimuli on indicated days after microinjection of control scrambled siRNA (Scr) or *Tet1* siRNA (Si) into the unilateral L4 and L5 DRGs on the ipsilateral side. *n*=8 rats group^−1^. *^∗∗^P*<0.01 *vs* the Scr group at the corresponding time point by two-way anova with repeated measures followed by *post hoc* Tukey test. (f)Levels of TET1, TET2 and TET3 in the microinjected rat L4 and L5 dorsal horn 7 days after microinjection of control scrambled siRNA (Scr) or *Tet1* siRNA (Si). *n*=3 rats group^−1^. *^∗^P*<0.05 by two-tailed unpaired Student *t* test. (g–i) Paw withdrawal threshold to mechanical stimuli (g) and paw withdrawal latencies to heat (h) and cold (i) stimuli on indicated days after microinjection of control scrambled siRNA (Scr) or *Tet1* siRNA (Si) into the unilateral L4 and L5 dorsal horn on the ipsilateral side. *n*=8 rats group^−1^. *^∗∗^P*<0.01 *vs* the Scr group at the corresponding time point by two-way anova with repeated measures followed by *post hoc* Tukey test.

To show whether TET1 downregulation in the DRG or dorsal horn from the REMSD-treated incisional rats is sufficient for the short-term sleep deprivation-induced exacerbation and prolongation of surgical pain, we mimicked this downregulation through microinjection of *Tet1* siRNA into the unilateral L4 and L5 DRGs or dorsal horn in naïve rats. The scrambled siRNA and vehicle were used as negative controls. To validate the specificity and selectivity of *Tet1* siRNA, we transfected siRNA into the cultured primary sensory neurones from the DRGs. *Tet1* siRNA, but not scrambled siRNA, reduced the level of TET1 by 68% compared with the vehicle group (Fig. 4a). Neither siRNA affected basal levels of TET2 and TET3 (Fig. 4a). As expected, the level of TET1, but not TET2 and TET3, in the ipsilateral L4 and L5 DRGs on day 7 after microinjection of *Tet1* siRNA into unilateral L4 and L5 DRGs decreased by 67% compared with the control scrambled siRNA-microinjected group (Fig. 4b). The *Tet1* siRNA-microinjected rats exhibited mechanical allodynia, shown by the reductions in paw withdrawal threshold to mechanical stimulation, and heat and cold hyperalgesia, evidenced by the reductions in paw withdrawal latencies to heat and cold stimuli on days 4 and 7 post-microinjection on the ipsilateral side (Fig. 4c–e). Microinjection of control scrambled siRNA into unilateral L4 and L5 DRGs did not alter basal responses to mechanical, heat and cold stimuli on the ipsilateral side (Fig. 4c–e). Similar results were observed after microinjection of *Tet1* siRNA or control scrambled siRNA into the unilateral L4 and L5 dorsal horn (Fig. 4f–i). Neither siRNA altered basal paw withdrawal responses to mechanical and heat stimuli on the contralateral side (supplementary Fig. 5) or normal locomotor activity (Supplementary Table 3).

siRNA can produce off-target effects. To further validate the aforementioned siRNA data, we genetically knocked down TET1 in the DRG through microinjection of AAV5-Cre into the unilateral L3 and L4 DRGs of *Tet1*^*f/f*^ mice. AAV5-GFP was used as a control. As predicted, the level of TET1 was reduced by 51% in the AAV5-Cre-microinjected DRGs compared to that in the AAV5-GFP-microinjected DRGs in *Tet1*^*f/f*^ mice (Supplementary Fig. 6a). AAV5-Cre-microinjected *Tet1*^*f/f*^ mice displayed the increases in paw withdrawal frequencies to 0.07 and 0.4 g von Frey filament stimuli and the decreases in paw withdrawal latencies to heat and cold stimuli on the ipsilateral (but not contralateral) side (Supplementary Fig. 6b–h). These nociceptive hypersensitivities occurred 3 weeks after viral microinjection and persisted for at least 5 weeks (Supplementary Fig. 6b–h). AAV5-GFP microinjection did not alter basal responses to mechanical, heat and cold stimuli on either side (Supplementary Fig. 6b–h). AAV5-Cre-microinjected *Tet1*^*f/f*^ mice also exhibited stimulation-independent spontaneous ongoing pain, evidenced by spending more time in the lidocaine-paired chamber (Supplementary Fig. 6i–j). All microinjected mice have normal locomotor activity (Supplementary Table 3). These findings indicate that TET1 knockdown in the DRG or dorsal horn can lead to surgical pain-like symptoms.

### TET1 downregulation is responsible for short-term sleep deprivation-induced reduction of MOR in DRG and dorsal horn

**Fig 5:**
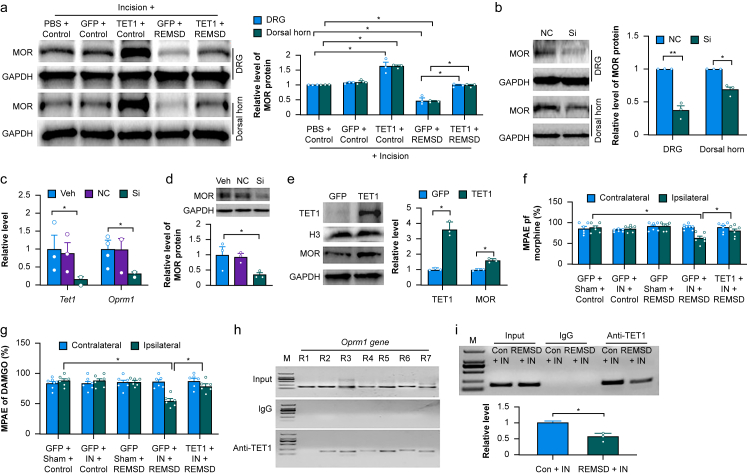
TET1 downregulation is responsible for short-term sleep deprivation-induced reduction of μ-opioid receptor in the dorsal root ganglion and dorsal horn of incisional rats. (a) Level of MOR in the ipsilateral L4 and L5 DRGs and dorsal horn on day 9 post-incision in the control- or REMSD-treated rats with microinjection of PBS, HSV-GFP (GFP) or HSV-TET1 (TET1) into these two regions, respectively, 3 days before incision. n=3 repeats (rats) group^−1^. *^∗^P*<0.05 by one-way anova followed by *post hoc* Tukey test. (b) Level of MOR in the microinjected rat L4 and L5 DRGs or dorsal horn 7 days after microinjection of control scrambled siRNA (Scr) or *Tet1* siRNA (Si). *n*=3 rats group^−1^. *^∗^P*<0.05, *^∗∗^P*<0.01 by two-tailed unpaired Student *t* test. (c, d) Levels of *Tet1* mRNA (c), *Oprm1* mRNA (c) and MOR protein (d) in the cultured DRG neurones with the treatments of vehicle (Veh), control scrambled siRNA (Scr) or *Tet1* siRNA. *n*=3 biological repeats group^−1^. *^∗^P*<0.05 by one-way anova followed by *post hoc* Tukey test. (e) Levels of TET1 and MOR in the cultured DRG neurones transduced with HSV-GFP (GFP) or HSV-TET1 (TET1). *n*=3 biological repeats group^−1^. *^∗^P*<0.05 by two-tailed unpaired Student *t* test. (f, g) Maximal possible analgesic effects (MPAEs) caused by intrathecal injection of morphine (7.5 μg; f) or DAMGO (0.5 μg, g) on the ipsilateral and contralateral sides on day 9 after incision or sham surgery in the control- or REMSD-treated rats with microinjection of HSV-GFP (GFP) or HSV-TET1 (TET1) into the ipsilateral L4 and L5 DRGs 3 days before incision. *n*=6 rats group^−1^. *^∗^P*<0.05 by two-way anova followed by *post hoc* Tukey test. (h) Rabbit anti-TET1 immunoprecipitated the region 2 (R2, -310∼-143), region 3 (R3, -164∼-6), region 4 (R4, -7∼+166), region 5 (R5, +142∼+312), region 6 (R6, +238∼+439), and region 7(R7: +391∼+558), but not region 1 (R1, -450∼-288), within the *Oprm1* gene in rat DRG. Input, total purified fragments. M, ladder marker. *n*=3 repeats. (i) The binding activity of TET1 to regions 3 within the *Oprm1* gene promoter in the ipsilateral L4 and L5 DRGs from control- or REMSD-treated rats on day 9 post-incision (IN). *n*=3 biological repeats group^−1^. ^∗^*P*<0.05 by 2-tailed unpaired Student *t* test.

Our previous studies revealed that MOR reduction in the DRG and dorsal horn contributed to short-term sleep deprivation-induced delay of surgical pain recovery.^14^^,^^20^ We examined whether TET1 downregulation was responsible for this reduction in the DRG and dorsal horn from the REMSD-treated incisional rats. Consistent with the previous study,^14^ the level of MOR in the ipsilateral L4 and L5 DRGs and dorsal horn from the HSV-GFP-microinjected REMSD rats on day 9 postincision was reduced by 63% and 54%, respectively, compared with the corresponding PBS-microinjected control rats that underwent paw incision (Fig. 5a). These reductions were abolished in the HSV-TET1-microinjected REMSD rats that received incision (Fig. 5a). In the HSV-TET1-microinjected control rats that underwent paw incision, the basal level of MOR in the ipsilateral L4 and L5 DRGs and dorsal horn on day 9 post-incision was elevated by 1.60-fold and 1.63-fold, respectively, compared with the corresponding PBS-microinjected control rats that received incision (Fig. 5a). As expected, the basal level of MOR in the ipsilateral L4 and L5 DRGs and dorsal horns from the HSV-GFP-microinjected control rats on day 9 after incision was unchanged (Fig. 5a). Conversely, the amount of MOR in the ipsilateral L4 and L5 DRGs and dorsal horn on day 7 after microinjection of *Tet1* siRNA were reduced by 63% and 32%, respectively, compared with the corresponding control scrambled siRNA-microinjected groups (Fig. 5b). Consistently, the transfection of *Tet1* siRNA into the cultured DRG neurones reduced the levels of both *Tet1* and *Oprm1* mRNAs by 57% and 45%, respectively (Fig. 5c) and the level of MOR protein by 70% (Fig. 5d). The transduction of HSV-TET1 into the cultured DRG neurones increased the amounts of TET1 and MOR by 2.37-fold and 1.61-fold, respectively (Fig. 5e). In addition, overexpression of MOR in the DRG through co-microinjection of a lentivirus expressing full-length *Oprm1* mRNA (LV-MOR) into the ipsilateral L4 and L5 DRGs rescued the *Tet1* siRNA–induced reduction of MOR in microinjected DRGs on day 7 post-microinjection (Supplementary Fig. 7a) and abolished *Tet1* siRNA–induced mechanical allodynia and heat and cold hyperalgesia on the ipsilateral side 4 and 7 days post-microinjection (Supplementary Fig. 7b). Similar results were observed after co-microinjection of LV-MOR with *Tet1* siRNA into the ipsilateral L4 and L5 dorsal horn (Supplementary Fig. 7c and d). Together, these data show that MOR is a critical downstream target of TET1 responsible for mediating its effects.

More importantly, we tested whether short-term sleep deprivation-caused TET1 downregulation in the DRG and dorsal horn affected MOR-mediated analgesic activity in the incisional rats. Morphine (7.5 μg^14^) was injected intrathecally on day 9 after incision in the control or REMSD-treated rats with pre-microinjection of HSV-TET1 or HSV-GFP into the ipsilateral L4 and L5 DRGs. The MPAE of morphine on the ipsilateral side of the HSV-GFP-microinjected REMSD rats on day 9 post-incision was significantly reduced by 28% of the value on the ipsilateral side of the HSV-GFP-microinjected control rats on day 9 post-sham surgery (Fig. 5f). This reduction was markedly rescued on the ipsilateral side of the HSV-TET1-microinjected REMSD and incision rats (Fig. 5f). As expected, morphine produced robust analgesia on the ipsilateral side of the HSV-GFP-microinjected control or REMSD rats 9 days post-sham surgery, on the ipsilateral side of the HSV-GFP-microinjected control rats 9 days post-incision and on the contralateral side of all treated groups (Fig. 5f). Similar observations were found after intrathecal injection of DAMGO, a highly selective MOR agonist (0.5 μg^14^), on day 9 postincision or sham surgery in the REMSD- or control-treated rats with pre-microinjection of HSV-GFP or HSV-TET1 (Fig. 5g). Together, these *in vivo* and *in vitro* results indicate that TET1 downregulation likely contributes to a MOR reduction in the DRG and dorsal horn under the conditions of short-term sleep deprivation-caused exacerbation and prolongation of surgical pain.

**Fig 6:**
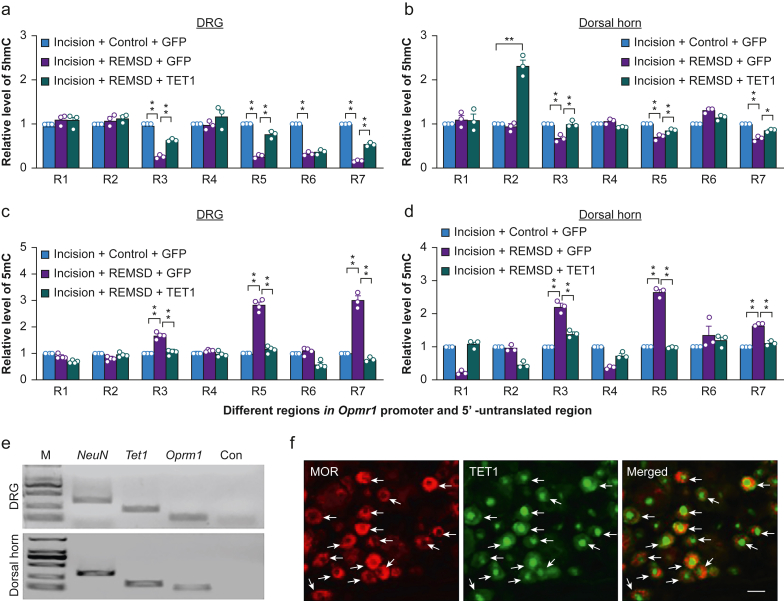
TET1 co-expresses with μ-opioid receptor in neurones of dorsal root ganglion and dorsal horn and rescues the REMSD-induced decrease of 5hmC and blocks the corresponding increase of 5mC within the *Oprm1* gene in the DRG and dorsal horn from incisional rats. (a, b) Level of 5hmC in seven regions (R1, -450∼-288; R2, -310∼-143; R3, -164∼-6; R4, -7∼+166; R5, +142∼+312; R6, +238∼+439; R7: +391∼+558) of the *Oprm1* gene in the ipsilateral L4 and L5 DRGs (a) and dorsal horn (b) from control- or REMSD-treated rats on day 9 postincision. *n*=3 biological repeats group^−1^. ^∗∗^*P*<0.01 by one-way anova followed by *post hoc* Tukey test. (c, d) Level of 5mC in seven regions (as indicated above) of the *Oprm1* gene in the ipsilateral L4 and L5 DRGs (c) and dorsal horn (d) from control- or REMSD-treated rats on day 9 postincision. *n*=3 biological repeats group^−1^. ^∗∗^*P*<0.01 by one-way anova followed by *post hoc* Tukey test. (e) Co-expression of *Tet1* mRNA with *Oprm1* mRNA in individual DRG neurones and dorsal horn neurones. M: ladder marker. Con: control distilled water. *n*=3 repeats. (f) Individual neurones double labelled by TET1 (in nucleus; green) and MOR (in cytoplasm; red) in the DRG (arrows). *n*=3 rats. Scale bar: 30 μm.

To further elucidate how TET1 downregulation was responsible for MOR reduction in the DRG and dorsal horn in the REMSD-treated incisional rats, we performed a ChIP assay and found that TET1 bound to 6 regions (R2: -310∼-143; R3: -164∼-6; R4: -7∼+166; R5: +142∼+312; R6: +238∼+439; R7: +391∼+558) of the *Oprm1* gene, as evidenced by the amplification of these regions (out of 7 regions from -450 to +558) from the complexes immunoprecipitated with the TET1 antibody in the nuclear fraction from naïve rat DRG (Fig. 5h). The binding activity of TET1 to R3 region in the ipsilateral L4 and L5 DRGs from the REMSD-treated group was reduced by 45% compared to the control group on day 9 after incision (Fig. 5i). Furthermore, the HSV-GFP-microinjected REMSD rats displayed a substantial increase in the level of 5mC and a corresponding decrease in the amount of 5hmC in the R3, R5 and R7 regions in the ipsilateral L4 and L5 DRGs and dorsal horn on day 9 postincision (Fig. 6a–d). However, in the ipsilateral L4 and L5DRGs and dorsal horn of the HSV-TET1-microinjected REMSD rats, 5mC increases in these regions were significantly blocked, and the corresponding 5hmC decreases were markedly rescued (Fig. 6a–d). Given that *Tet1* mRNA co-expresses with *Oprm1* mRNA in the individual neurones of the DRG and dorsal horn and that approximately 93% of TET1-labelled neurones are MOR-positive in the DRG (Fig. 6e–f), these findings suggest that, under the conditions of incision, short-term sleep deprivation-caused TET1 downregulation leads to a MOR reduction likely because of a loss of TET1 binding to the *Oprm1* promoter, resulting in an increase of 5mC and a corresponding decrease of 5hmC in the *Oprm1* promoter and consequent silence of *Oprm1* gene expression in the DRG and dorsal horn.

## Discussion

Surgical patients, particularly in the intensive care unit, often experience short-term sleep disruption, which results in increased pain perception, lowered pain threshold and delayed surgical pain recovery.^10^, ^11^, ^12^, ^13^ Understanding the mechanisms of how short-term sleep disruption exacerbates and prolongs surgical pain may provide optimal and efficient interventions for postsurgical pain. The present study carried out a preclinical rat model of postoperative short-term REMSD to mimic the sleep disruption-induced increase of surgical pain in patients^14^^,^^20^ and reported that REMSD postincision not only exacerbated and prolonged surgical pain but also downregulated TET1 expression in neurones of the DRG and SDH. More importantly, rescuing this downregulation, respectively, in these two regions blocked the REMSD-induced exacerbation and prolongation of incisional pain, likely by restoring a reduction of MOR in the DRG or SDH. TET1 downregulation in the DRG or SDH may be required for sleep disturbance to delay surgical pain recovery.

Previous studies from clinical observations revealed that chronic sleep deprivation increased basal pain perception in healthy volunteers.^31^^,^^32^ Preclinical observations also showed that long-term intermittent or consecutive REMSD augmented behavioural responses to noxious heat and electrical stimuli in naïve animals.^31^^,^^33^ However, in clinical settings, surgical patients often experience normal pain perception before surgery, although they have short-term sleep disturbance at various degrees throughout the pre and postoperative periods. This postoperative sleep disturbance heightens acute postoperative pain and increases analgesic consumption.^34^^,^^35^ To mimic this clinical condition, we recently developed a preclinical rat model in which short-term sleep disturbance induced by REMSD for 6 h daily for 3 consecutive days did not affect basal pain perception but prolonged incisional pain without significant sex difference.^14^^,^^20^ The present study first verified that short-term REMSD exacerbated and prolonged evoked nociceptive hypersensitivity after incision and then further showed that short-term REMSD also led to stimulation-independent ongoing pain in male rats. Notably, we reported hyperactivity in small (but not medium and large) DRG neurones and neuronal and astrocyte hyperactivity in the SDH from the REMSD-treated rats on day 9 post-incision; at this time point, control rats displayed the complete recovery of incisional pain. These data further validate the preclinical animal model of REMSD-induced delay of surgical pain recovery.

TET1 can be regulated under pathological pain conditions. Consistent with previous reports,^36^^,^^37^ TET1 is expressed exclusively in neurones of the DRG and SDH. Peripheral nerve injury or inflammation upregulated TET1 in the ipsilateral spinal cord, but not in DRG.^24^^,^^36^, ^37^, ^38^, ^39^ Systemic injection of the chemotherapy drug oxaliplatin increased the level of TET1 in the spinal cord.^40^ The present study reported the downregulation of TET1 in neurones of the ipsilateral L4 and L5 DRGs and SDH from incisional rats that underwent REMSD. The changes of TET1 expression are significantly correlated with distinct aetiologies under different pathological conditions. Interestingly, neither incision nor REMSD alone altered basal expression of TET1 in these two regions. It appears that both incision-induced noxious input and short-term REMSD are necessary for TET1 downregulation. The mechanisms underlying the interaction between incision and REMSD leading to this downregulation remain unknown. Considering that REMSD did not change basal expression of TET2, TET3, DNMT1, DNMT3a and DNMT3b in the DRG and SDH after incision, TET1 downregulation in the DRG and SDH is REMSD- and incision-specific. The mechanisms underlying this isoform-specific downregulation may be related to the REMSD- and incision-induced stress. The TET1 promoter contains regulatory elements that are particularly vulnerable to glucocorticoid signalling, inflammatory mediators, circadian disruption and oxidative stress.^41^, ^42^, ^43^ The REMSD-treated incisional rats displayed an increase in immobility duration, a loss of interest in pleasure and an elevation of corticosterone in the blood.^14^ Intrathecal injection of a selective glucocorticoid receptor antagonist RU38486, or bilateral adrenalectomy completely abolished the REMSD-induced delay in postsurgical pain recovery.^14^ These findings strongly suggest that glucocorticoid signalling is involved in the REMSD- and incision-induced TET1 downregulation in the DRG and SDH. It is possible that the promoters of TET2, TET3 and DNMTs have distinct regulatory elements that do not respond to glucocorticoid-triggered stress. Indeed, DNMT1, DNMT3a and DNMT3b are housekeeping methyltransferases essential for cellular survival.^44^ Their expression is tightly buffered and resistant to acute stress.^45^ TET2 and TET3 maintain baseline demethylation and are more strongly regulated at the post-translational level.^46^^,^^47^ Other potential mechanisms underlying TET1-specific downregulation in the DRG and DH under the REMSD plus incision conditions remain to be investigated in future studies.

TET1 downregulation in neurones of the DRG or SDH is required for postoperative short-term REMSD to delay the recovery of surgical pain. Rescuing TET1 downregulation in the ipsilateral L4 and L5 DRGs or SDH through microinjection of HSV-TET1 into these two regions, respectively, abolished the REMSD-caused delay of surgical pain recovery. Knocking down TET1 expression in the DRG or SDH through microinjection of *Tet1* siRNA led to surgical pain-like symptoms in rats. Consistent with a recent study,^48^ genetic knockdown of TET1 in the unilateral L3 and L4 DRGs through microinjection of AAV5-Cre into these DRGs of *Tet1*^*f/f*^ mice produced augmented responses to mechanical, heat and cold stimuli on the ipsilateral side. This study focused exclusively on male rats. Sex-specific TET1 downregulation-mediated epigenetic regulation on pain behaviours cannot be excluded and warrants future study, although earlier studies suggest minimal sex differences in REMSD-induced prolongation of surgical pain between male and female rats.^14^^,^^20^ In addition, given that sleep deprivation is a systemic insult, REMSD-induced TET1 downregulation may occur in other brain regions of incisional rats. The contribution of TET1 downregulation in these brain regions to the exacerbation and prolongation of surgical pain could not be ruled out. These expectations will be examined in our future studies.

We further showed that MOR reduction in the DRG and SDH may be involved in the contribution of TET1 downregulation in these two regions to the REMSD-caused delay of surgical pain recovery. In line with the previous study,^14^ short-term REMSD led to a substantial reduction of MOR in the DRG and SDH on day 9 post-incision; at this time point, short-term REMSD downregulated TET1 in these two regions and exacerbated incisional pain. Rescuing TET1 downregulation in the ipsilateral L4 and L5 DRGs and SDH, respectively, reversed the REMSD-induced reductions of MOR expression in the corresponding region and MOR-mediated analgesic effect in incisional rats. TET1 overexpression in the DRG and SDH, respectively, upregulated basal expression of MOR in the corresponding region in the HSV-TET1-microinjected control rats with incision. This upregulation may interpret an increase in the resolution of incision-induced mechanical hypersensitivity in the HSV-TET1-microinjected control group. TET1 knockdown in *in vitro* cultured DRG neurones and *in vivo* DRG and SDH resulted in the decreases in the levels of *Oprm1* mRNA and MOR protein. Overexpression of MOR in the DRG or SDH rescued the TET1 knockdown–induced reduction of MOR in the corresponding region and abolished TET1 knockdown–induced nociceptive hypersensitivity. Moreover, the binding activity of TET1 to the *Oprm1* promoter was reduced in the DRG and SDH from the REMSD-treated rats on day 9 postincision. Rescuing TET1 downregulation in the DRG and SDH blocked the REMSD-induced increase of 5-mC and attenuated the REMSD-induced decrease of 5-hmC in the *Oprm1* promoter in these two regions on day 9 post-incision. These findings indicate that TET1 downregulation disrupts the dynamic equilibrium between 5-mC and 5-hmC in the *Oprm1* promoter, resulting in increased 5-mC (e.g. DNA methylation) despite the absence of expressional changes of DNMTs in the DRG and SDH from the REMSD-treated incisional rats. Given that DNA methylation suppresses gene transcription,^17^ TET1 downregulation is likely responsible for MOR reduction through elevating DNA methylation in the *Oprm1* promoter in the DRG and SDH from the REMSD-treated incisional rats.

Interestingly, nerve trauma–induced MOR downregulation in injured DRG neurones is likely mediated by increased DNMT3a, as DRG-specific knockdown of this enzyme rescued MOR expression by suppressing promoter DNA hypermethylation following injury.^49^ It appears that different pathological conditions drive DNA methylation–dependent MOR downregulation through distinct epigenetic mechanisms. Considering that the endogenous opioids acting on MOR exert tonic inhibitory effects on nociceptive information transmission and that TET1 co-expresses with MOR in neurones of DRG and SDH, TET1 downregulation-induced reduction of MOR in the DRG and SDH may lead to a loss of tonic inhibitory effect, resulting in exacerbation of postsurgical pain. Although direct evidence linking TET1-mediated demethylation of the MOR locus in human tissues or clinical datasets is currently lacking, clinical studies have shown that DNA methylation at the human *OPRM1* promoter is altered in opioid dependence and other conditions.^50^ Establishing such a link would strengthen the translational relevance of our findings and represents an important direction for future research. It should be noted that, besides MOR, short-term REMSD plus incision may produce changes in the expression of other pain-associated receptors and ion channels (e.g. kappa opioid receptor^14^) and sleep-related genes (e.g. clock and Bmal1^51^) in the DRG and SDH and that these changes may also occur in pain-associated brain regions. These potential mechanisms cannot be ruled out and will be further studied.

In summary, the current study explored a novel molecular mechanism by which TET1 downregulation-induced MOR reduction in the DRG and SDH contributes to the role of postoperative short-term sleep disturbance in the exacerbation and prolongation of surgical pain. Rescuing downregulated TET1 has been shown to abolish short-term sleep disturbance-induced delay of postsurgical pain recovery, without affecting acute or basal pain and locomotor activity. TET1 is likely a potential target for treatment of this disorder. Nevertheless, other unwanted adverse effects should be paid more attention, as TET1 is expressed widely in body tissues.

## Authors’ contributions

Study conception and design: JC, PKW, BX, XG, YXT

Data acquisition and analysis: JC, PKW, BX, BX, XG, WC, ZP, YXT

Drafting figures: JC, PKW, BX, XG, WC, SS, ZL, JD, AB, HH, YXT

Drafting manuscript: JC, YXT

Finalising manuscript: YXT

## Funding

This work was supported by US NIH grants (R01 NS111553, R01 NS117484 and DK142785). All data are available upon request.

## Declaration of interests

The authors declare that they have no conflicts of interest.

## Figures and Tables

**Figure:**
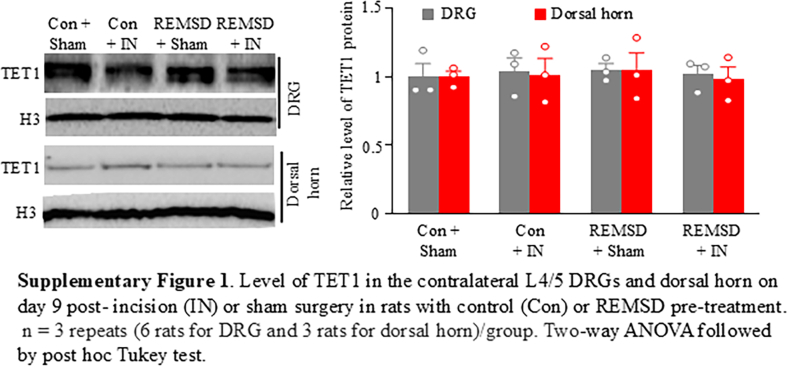


**Figure:**
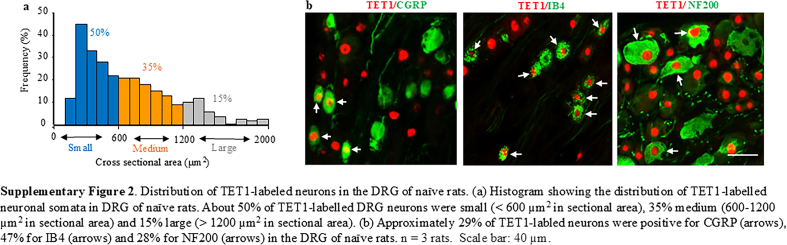


**Figure:**
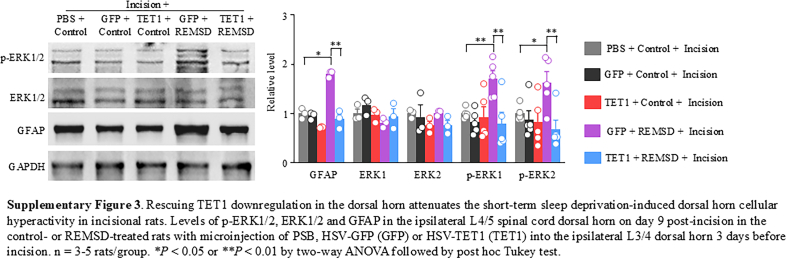


**Figure:**
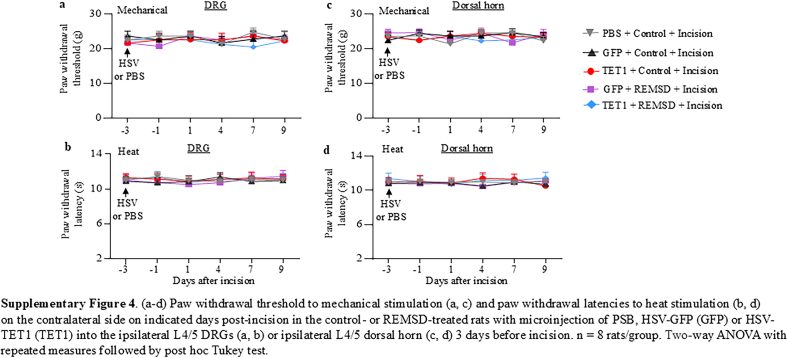


**Figure:**
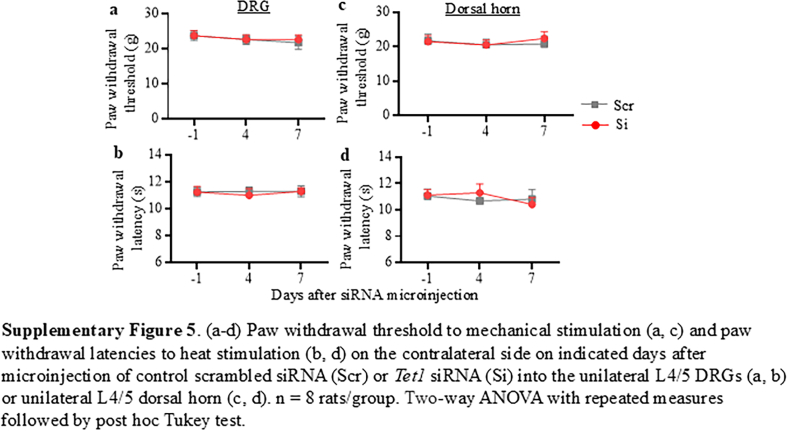


**Figure:**
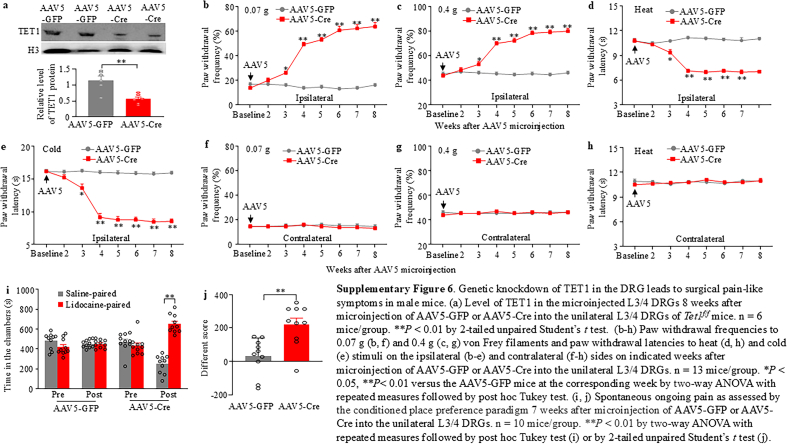


**Figure:**