# Supplementary material for: TET1 downregulation in the dorsal root ganglion and spinal cord is required for short-term sleep disturbance to delay surgical pain recovery
Source: Br J Anaesth. 2026 May 5;136(6):1910–24. doi: 10.1016/j.bja.2026.02.025 (PMC13197928; doi:10.1016/j.bja.2026.02.025)
Supplement: multimedia component 1 [file mmc1.doc]

| Purpose | Names | Sequences(5’-3’) |
| --- | --- | --- |
| RT-PCR | *Oprm1-F* | 5’-TTCTTGTTTCAGATACGCGGA-3’ |
|  | *Oprm1-R* | 5’-GGTCGGTGTTTTCATCAGTTAGG-3’ |
|  | *Tet1-F* | 5’-TGTCACCTGTTGCATGGATT-3’ |
|  | *Tet1-R* | 5’-TTGGATCTTGGCTTTCATCC-3’ |
|  | *Gapdh-F* | 5’-TCGGTGTGAACGGATTTGGC-3’ |
|  | *Gapdh-R* | 5’-CCTTCAGGTGAGCCCCAGC-3’ |
| siRNA | *Tet1* siRNA | 5′-UUGUUGAGGGUACAUCUUCTT-3′ |
|  | Scrambled siRNA | 5′-GAAGAUGUACCCUCAACAATT-3′ |
| Vector construc-  tion | *Tet1-F* (1st fragment) | 5’-CCACACCTCATGTGACCAAG-3’ |
|  | *Tet1-R* (1st fragment) | 5’-CTGTCTTGGATGGGGAGGAG-3’ |
|  | *Tet1-F* (2^nd^ fragment) | 5’-CACCTGTAAGAAACGGCATGAA-3’ |
|  | *Tet1-R* (2^nd^ fragment) | 5’-TGATGGCAATGATTGGAAGA-3’ |
|  | *Tet1-n-F* (1st fragment) | 5’-CACCACCATGTCTCGGTCCCGCC-3’ |
|  | *Tet1-n-R* (1st fragment) | 5’-CTGTCTTGGATGGGGAGGAG-3’ |
|  | *Tet1-n-F* (2^nd^ fragment) | 5’-CACCTGTAAGAAACGGCATGAA-3’ |
|  | *Tet1-n-R* (2^nd^ fragment) | 5’-ATTACGTGAGCGGTTTCTACA-3’ |
| Single cell RT-PCR | *Tet1-F* | 5’-TGTCACCTGTTGCATGGATT-3’ |
|  | *Tet1-R* | 5’-TTGGATCTTGGCTTTCATCC-3’ |
|  | *Oprm1-F* | 5’-TTCCTGGTCATGTATGTGATTGTA-3’ |
|  | *Oprm1-R* | 5’-GGCAGTGTACTGGTCGCTAA-3’ |
|  | *Gapdh-F* | 5’-TCGGTGTGAACGGATTTGGC-3’ |
|  | *Gapdh-R* | 5’-CCTTCAGGTGAGCCCCAGC-3’ |
| ChIP-PCR | *Oprm1-ChIP-F1* | 5’-TTCTTGTTTCAGATACGCGGA-3’ |
|  | *Oprm1-ChIP-R1* | 5’-GGTCGGTGTTTTCATCAGTTAGG-3’ |
|  | *Oprm1-ChIP-F2* | 5’-CTAACTGATGAAAACACCGACCT-3’ |
|  | *Oprm1-ChIP-R2* | 5’-GAGAGTCAGCCTCCTCGGTC-3’ |
|  | *Oprm1-ChIP-F3* | 5’-CGACCGAGGAGGCTGACTCT-3’ |
|  | *Oprm1-ChIP-R3* | 5’-CTTAGAAGTACACAGAGGCGCAT-3’ |
|  | *Oprm1-ChIP-F4* | 5’-AGGTGGGAGGGGGCTACAAG-3’ |
|  | *Oprm1-ChIP-R4* | 5’-GAGCACTCAGACTTTTCGGGT-3’ |
|  | *Oprm1-ChIP-F5* | 5’-GAACCCGAAAAGTCTGAGTGC-3’ |
|  | *Oprm1-ChIP-R5* | 5’-AACGTGGGACAAGTTGAGCC-3’ |
|  | *Oprm1-ChIP-F6* | 5’-GCGACTGCTCAGACCCCTTA-3’ |
|  | *Oprm1-ChIP-R6* | 5’-CGATAGAGTAGAGGGCCATGATG-3’ |
|  | *Oprm1-ChIP-F7* | 5’-AGCCCTTCCATGGTCACAGC-3’ |
|  | *Oprm1-ChIP-R7* | 5’-TCCTCTGTCACTCTCGGGCT-3’ |

**Supplementary Table 1**. Primers and siRNAs

**Supplementary Table 2**. Effect of REMSD on membrane input resistance, and other action potential parameters in small, medium and large DRG neurons of rats on day 9 post-incision

|  | Large neurons | | | Medium neurons | | | Small neurons | | |
| --- | --- | --- | --- | --- | --- | --- | --- | --- | --- |
|  | REMSD + IN | Control + IN | t/p value | REMSD + IN | Control + IN | t/p value | REMSD + IN | Control + IN | t/p value |
| n | 32 cells, 8 rats | 26 cells, 7 rats |  | 30 cells, 8 rats | 24 cells, 7 rats |  | 31 cells, 8 rats | 26 cells, 7 rats |  |
| R_in_, MΩ | 40.10 ± 2.79 | 39.99 ± 3.27 | -0.031/0.975 | 46.20 ± 2.37 | 45.17 ± 2.22 | 0.317/0.753 | 57.40 ± 3.51 | 56.11 ± 2.74 | -0.277/0.783 |
| APT, mV | -13.50 ± 0.91 | -13.14 ± 0.69 | 0.287/0.775 | -13.20 ± 0.66 | -13.23 ± 0.63 | -0.010/0.992 | -12.50 ± 0.68 | -12.96 ± 0.75 | -0.426/0.672 |
| APO, mV | 42.70 ± 2.06 | 42.50 ± 1.36 | -0.088/0.930 | 46.20 ± 1.78 | 45.98 ± 1.65 | -0.079/0.938 | 46.60 ± 1.53 | 40.72 ± 1.26 | -2.888/0.006 |
| APA, mV | 101.10 ± 2.14 | 100.69 ± 1.58 | -0.138/0.891 | 103.80 ± 1.97 | 103.66 ± 1.74 | 0.068/0.946 | 98.30 ± 1.86 | 97.67 ± 1.08 | -0.296/0.768 |
| AHPA, mV | -14.80 ± 0.67 | -15.04 ± 0.88 | -0.222/0.825 | -15.40 ± 0.99 | -15.89 ± 1.17 | 0.297/0.768 | -16.00 ± 0.88 | -16.25 ± 0.97 | -0.186/0.853 |

Values are mean ± SEM. IN: incision. R_in_: membrane input resistance. APA: action potential amplitude. APT: action potential threshold. APO: action potential overshoot. AHPA: afterhyperpolarization.

**Supplementary Table 3.** Locomotor function.

| Treatment groups | | Placing | | | Grasping | | | | Righting | |
| --- | --- | --- | --- | --- | --- | --- | --- | --- | --- | --- |
| Rats | | |  |  | | | |  | | |
| Control + Sham | | | 5 (0) | | | | 5 (0) | | | 5 (0) |
| Control + IN | | | 5 (0) | | | | 5 (0) | | | 5 (0) |
| REMSD + Sham | | | 5 (0) | | | | 5 (0) | | | 5 (0) |
| REMSD + IN | | | 5 (0) | | | | 5 (0) | | | 5 (0) |
| PBS (DRG) + Control + Incision | | | 5 (0) | | | | 5 (0) | | | 5 (0) |
| GFP (DRG) + Control + Incision | | | 5 (0) | | | | 5 (0) | | | 5 (0) |
| TET1 (DRG) + Control + Incision | | | 5 (0) | | | | 5 (0) | | | 5 (0) |
| GFP (DRG) + REMSD + Incision | | | 5 (0) | | | | 5 (0) | | | 5 (0) |
| TET1 (DRG) + REMSD + Incision | | | 5 (0) | | | | 5 (0) | | | 5 (0) |
| PBS (Dorsal horn) + Control + Incision | | | 5 (0) | | | | 5 (0) | | | 5 (0) |
| GFP (Dorsal horn) + Control + Incision | | | 5 (0) | | | | 5 (0) | | | 5 (0) |
| TET1 (Dorsal horn) + Control + Incision | | | 5 (0) | | | | 5 (0) | | | 5 (0) |
| GFP (Dorsal horn) + REMSD + Incision | | | 5 (0) | | | | 5 (0) | | | 5 (0) |
| TET1 (Doral horn) + REMSD + Incision | | | 5 (0) | | | | 5 (0) | | | 5 (0) |
| Control scrambled siRNA (DRG) | | | 5 (0) | | | | 5 (0) | | | 5 (0) |
| *Tet1* siRNA (DRG) | | | 5 (0) | | | | 5 (0) | | | 5 (0) |
| Control scrambled siRNA (Dorsal horn) | | | 5 (0) | | | | 5 (0) | | | 5 (0) |
| *Tet1* siRNA (Dorsal horn) | | | 5 (0) | | | | 5 (0) | | | 5 (0) |
| Mice |  | | | | |  | | | |  |
| AAV5-GFP (DRG) | | | 5 (0) | | | | 5 (0) | | | 5 (0) |
| AAV5-Cre (DRG) | | | 5 (0) | | | | 5 (0) | | | 5 (0) |
|  | | |  | | | |  | | |  |

n = 5-13 rats per group; five trials; Mean (SEM). IN: Incision. REMSD: rapid eye movement sleep deprivation. PBS: Phosphate-buffered saline. GFP: Enhanced green fluorescent protein. TET1: ten-eleven translocation methylcytosine dioxygenase 1. Si: small interfering RNA. AAV5: Adeno-associated virus serotype 5. Cre: Cre recombinase.
